# Pituitary Society Delphi Survey: An international perspective on endocrine management of patients undergoing transsphenoidal surgery for pituitary adenomas

**DOI:** 10.1007/s11102-021-01170-3

**Published:** 2021-07-20

**Authors:** Nicholas A. Tritos, Pouneh K. Fazeli, Ann McCormack, Susana M. Mallea-Gil, Maria M. Pineyro, Mirjam Christ-Crain, Stefano Frara, Artak Labadzhyan, Adriana G. Ioachimescu, Ilan Shimon, Yutaka Takahashi, Mark Gurnell, Maria Fleseriu, for the “Pituitary Society Delphi Collaborative Group”, Irina Bancos, Martin Bidlingmaier, Nienke Biermasz, Cesar Luiz Boguszewski, Jessica Brzana, John Carmichael, Philippe Chanson, Andjela Drincic, Yuval Eisenberg, Hidenori Fukuoka, Monica Gadelha, Luma Ghalib, Murray Gordon, Yona Greenman, Francisco Guarda, Miguel Hinojosa-Amaya, Ken Ho, Mirela-Diana Ilie, Niki Karavitaki, Larry Katznelson, Fahrettin Keleştimur, Andre Lacroix, Fabienne Langlois, Dawn Lim, Sebastian Neggers, Dan Niculescu, Stephan Petersenn, Rosario Pivonello, Gerald Raverot, Richard Ross, Roberto Salvatori, Carla Scaroni, Ismat Shafiq, Susmeeta Sharma, Antoine Tabarin, Stylianos Tsagarakis, Elena Valassi, Greisa Vila, Maggie Wierman

**Affiliations:** 1 Massachusetts General Hospital/Harvard Medical School, Boston, MA USA; 2 University of Pittsburgh School of Medicine, Pittsburgh, PA USA; 3 Garvan Institute of Medical Research, Darlinghurst, Australia; 4 Hospital Militar Central, Buenos Aires, Argentina; 5 Hospital de Clinicas, Facultad de Medicina, Universidad de la Republica, Montevideo, Uruguay; 6 University Hospital Basel, Basel, Switzerland; 7 Università Vita-Salute San Raffaele, Milano, Italy; 8 Cedars-Sinai Medical Center, Los Angeles, CA USA; 9 Emory University School of Medicine, Atlanta, GA USA; 10 Rabin Medical Center, Petah Tikva, Israel; 11 Nara Medical University, Nara, Japan; 12 Wellcome-MRC Institute of Metabolic Science, University of Cambridge and NIHR Cambridge Biomedical Research Centre, Addenbrooke’s Hospital, Box 289, Cambridge, CB2 0QQ UK; 13 Oregon Health & Science University, Portland, OR USA

**Keywords:** Delphi process, Pituitary adenoma, Transsphenoidal surgery, Hypopituitarism, Perioperative, Postoperative assessment

## Abstract

**Purpose:**

In adults and children, transsphenoidal surgery (TSS) represents the cornerstone of management for most large or functioning sellar lesions with the exception of prolactinomas. Endocrine evaluation and management are an essential part of perioperative care. However, the details of endocrine assessment and care are not universally agreed upon.

**Methods:**

To build consensus on the endocrine evaluation and management of adults undergoing TSS, a Delphi process was used. Thirty-five statements were developed by the Pituitary Society’s Education Committee. Fifty-five pituitary endocrinologists, all members of the Pituitary Society, were invited to participate in two Delphi rounds and rate their extent of agreement with statements pertaining to perioperative endocrine evaluation and management, using a Likert-type scale. Anonymized data on the proportion of panelists’ agreeing with each item were summarized. A list of items that achieved consensus, based on predefined criteria, was tabulated.

**Results:**

Strong consensus (≥ 80% of panelists rating their agreement as 6–7 on a scale from 1 to 7) was achieved for 68.6% (24/35) items. If less strict agreement criteria were applied (ratings 5–7 on the Likert-type scale), consensus was achieved for 88% (31/35) items.

**Conclusions:**

We achieved consensus on a large majority of items pertaining to perioperative endocrine evaluation and management using a Delphi process. This provides an international real-world clinical perspective from an expert group and facilitates a framework for future guideline development. Some of the items for which consensus was not reached, including the assessment of immediate postoperative remission in acromegaly or Cushing’s disease, represent areas where further research is needed.

## Introduction

Pituitary adenomas are common benign sellar masses and account for approximately 90% of sellar lesions in surgical case series [1–4]. In addition to pituitary adenomas, a large variety of other pathologic entities may occur in the sella. Transsphenoidal surgery is the cornerstone of management for large non-functioning sellar lesions as well as most non-prolactinoma functioning tumors.

Endocrine evaluation is essential in patients undergoing transsphenoidal surgery. Society guidelines have been published regarding the evaluation and management of non-functioning and functioning pituitary tumors [5–12]. However, there are still considerable uncertainties regarding the optimal endocrine assessment and management of these patients.

The Delphi process has been used with the goal of achieving consensus among experts from different countries on a variety of health-related topics using an iterative process, during which anonymized opinions from experts are fed back to the same expert panel in a series of rounds [13, 14].

In the present study, we sought to engage pituitary endocrinologists towards achieving consensus regarding the endocrine evaluation and management of patients undergoing TSS using a Delphi process.

## Methods

A Steering Committee, composed of members of the Pituitary Society Education Committee, developed the study objectives and the statement questions (please find statement questions summarized in Table 1). References from the literature were compiled. A total of 55 endocrinologists from 5 continents (Asia, Europe, North America, Oceania, South America), all members of the international Pituitary Society, were invited to participate in the Delphi process via electronic mail. Ethics approval was not required (the study did not enroll any patients and does not report on patient data).

**Table 1:** Proportion of panelists indicating some or complete agreement (rating 6 or 7 on a Likert-type scale) with individual items pertaining to the evaluation and management of adult patients undergoing transsphenoidal surgery

Item number	Item	First Delphi round	Second Delphi round
1	Patients with an apparent pituitary adenoma should have serum prolactin measured preoperatively	49/50 (98%)	Consensus achieved in Round 1
2	Patients with an apparent macroadenoma and minimally elevated prolactin levels should have serum prolactin measured in serial dilution preoperatively	31/50 (62%)	36/51 (70.6%)
3	Serum IGF-I should be measured in all patients with a sellar mass preoperatively	43/50 (86%)	Consensus achieved in Round 1
4	Patients with a macroadenoma or other large (≥ 1 cm) sellar mass should undergo evaluation for hypoadrenalism preoperatively	43/50 (86%)	Consensus achieved in Round 1
5	Patients with a sellar mass who present with symptoms or signs suggestive of hypoadrenalism should undergo evaluation of adrenal reserve preoperatively regardless of lesion size	43/50 (86%)	Consensus achieved in Round 1
6	All patients with an apparent pituitary adenoma and any symptoms or signs of hypercortisolemia should be evaluated for Cushing’s disease preoperatively	48/50 (96%)	Consensus achieved in Round 1
7	Thyroid function should be tested in all patients with a sellar mass preoperatively	42/50 (84%)	Consensus achieved in Round 1
8	Evaluation of gonadal function is advisable in patients with symptoms suggestive of hypogonadism, those with a large (≥ 1 cm) sellar mass and those with a functioning tumor regardless of size preoperatively	44/50 (88%)	Consensus achieved in Round 1
9	Evaluation of possible diabetes insipidus is advisable in patients with a sellar mass who present with polyuria and/or hypernatremia preoperatively	45/50 (90%)	Consensus achieved in Round 1
10	Patients with hypoadrenalism should receive glucocorticoid replacement preoperatively	48/49 (97.9%)	Consensus achieved in Round 1
11	Patients with hypothyroidism should receive thyroid hormone replacement preoperatively	40/50 (80%)	Consensus achieved in Round 1
12	Sex steroid replacement is advisable in symptomatic patients with central hypogonadism preoperatively	9/50 (18%)	9/45 (20%)
12a	Sex steroid replacement may be considered in symptomatic patients with central hypogonadism preoperatively	Question 12 modification for Round 2	27/51 (52.9%)
13	Preoperative medical therapy should be considered in patients with somatotropin-secreting adenomas	7/50 (14%)	8/46 (17.4%)
13a	Preoperative medical therapy may be considered in patients with somatotropin-secreting adenomas	Question 13 modification for Round 2	32/50 (64%)
14	Preoperative medical therapy should be considered in patients with corticotropin-secreting adenomas	13/50 (26%)	15/47 (31.9%)
14a	Preoperative medical therapy may be considered in patients with corticotropin-secreting adenomas	Question 14 modification for Round 2	37/51 (72.5%)
15	Stress dose glucocorticoid administration is advisable perioperatively	27/50 (54%)	26/49 (53.1%)
15a	Stress dose glucocorticoid administration is generally advisable perioperatively in patients with known or suspected adrenal insufficiency	Question 15 modification for Round 2	48/50 (96%)
16	Serum sodium should be monitored postoperatively	47/50 (94%)	Consensus achieved in Round 1
17	Morning serum cortisol should be monitored postoperatively (during an interval of 1–5 days postoperatively)	41/50 (82%)	Consensus achieved in Round 1
18	Full evaluation of pituitary function should be conducted 6–12 weeks after transsphenoidal surgery	46/49 (93.9%)	Consensus achieved in Round 1
19	Serum prolactin should be measured postoperatively (on postoperative day 1 or 2) in patients with presumed prolactin-secreting adenomas to evaluate for remission	27/49 (55.1%)	27/47 (57.4%)
19a	Serum prolactin may be measured postoperatively (on postoperative day 1 or 2) in patients with presumed prolactin-secreting adenomas to evaluate for remission	Question 19 modification for Round 2	38/51 (74.5%)
20	Dynamic testing should be obtained to evaluate the pituitary adrenal axis at 6–12 weeks postoperatively, if morning serum cortisol is not sufficiently high to assure sufficient adrenal function early postoperatively	43/49 (87.7%)	Consensus achieved in Round 1
21	Thyroid function should be assessed in all patients postoperatively (at 6–8 weeks)	46/49 (93.9%)	Consensus achieved in Round 1
22	Gonadal function should be evaluated in patients (6–12 weeks) postoperatively, including women of premenopausal age and men	44/49 (89.8%)	Consensus achieved in Round 1
23	Dynamic testing should be obtained to assess GH secretion postoperatively in patients with clinical suspicion of GH deficiency	34/49 (69.4%)	31/46 (67.4%)
23a	Dynamic testing may be obtained to assess GH secretion postoperatively in patients with clinical suspicion of GH deficiency	Question 23 modification for Round 2	43/51 (84.3%)
24	In patients with acromegaly, morning serum GH should be obtained early postoperatively (postoperative day 1 or 2) to evaluate endocrine remission	22/48 (45.8%)	24/48 (50%)
24a	In patients with acromegaly, morning serum GH may be obtained early postoperatively (postoperative day 1 or 2) to evaluate endocrine remission	Question 24 modification for Round 2	36/50 (72%)
25	Serum IGF-I should be obtained to evaluate endocrine remission at 6 weeks postoperatively. If elevated, serum IGF-I should be rechecked at 12 weeks postoperatively to document persistent disease activity before making treatment decisions	39/48 (81.1%)	Consensus achieved in Round 1
26	A glucose tolerance test should be obtained to evaluate endocrine remission of acromegaly several weeks postoperatively	26/49 (53.1%)	30/47 (63.8%)
26a	A glucose tolerance test may be obtained to evaluate endocrine remission of acromegaly several weeks postoperatively	Question 26 modification for Round 2	37/51 (72.5%)
27	Patients with acromegaly who are in endocrine remission should be evaluated biochemically for recurrence annually (or sooner if clinically indicated)	43/49 (87.7%)	Consensus achieved in Round 1
28	In patients with Cushing’s disease, endocrine testing should be conducted during the first postoperative week to document endocrine remission	41/48 (85.4%)	Consensus achieved in Round 1
29	In patients with Cushing’s disease, serum cortisol should be monitored to document endocrine remission postoperatively	44/48 (91.7%)	Consensus achieved in Round 1
30	In patients with Cushing’s disease, monitoring of plasma ACTH levels should be considered to document endocrine remission postoperatively	21/48 (43.7%)	14/47 (29.8%)
30a	In patients with Cushing’s disease, monitoring of plasma ACTH levels may be considered to document endocrine remission postoperatively	Question 30 modification for Round 2	32/51 (62.7%)
31	In patients with Cushing’s disease, 24 h urinary free cortisol should be monitored to document endocrine remission postoperatively	19/48 (39.6%)	25/48 (52%)
31a	In patients with Cushing’s disease, 24 h urinary free cortisol may be monitored to document endocrine remission postoperatively	Question 31 modification for Round 2	40/51 (78.4%)
32	In patients with Cushing’s disease, late night salivary cortisol should be monitored to document endocrine remission postoperatively	25/48 (52.1%)	30/47 (63.8%)
32a	In patients with Cushing’s disease, late night salivary cortisol may be monitored to document endocrine remission postoperatively	Question 32 modification for Round 2	38/51 (74.5%)
33	Patients with Cushing’s disease who are in endocrine remission should be evaluated for recurrence annually (or sooner if clinically indicated)	45/48 (93.7%)	Consensus achieved in Round 1
34	Patients with an apparent clinically non-functioning pituitary adenoma may be evaluated for Cushing’s disease preoperatively	Question added for Round 2	32/46 (69.6%)
35	Desmopressin may be administered “on demand” (as required) in patients who underwent transsphenoidal surgery and developed central diabetes insipidus in the postoperative period	Question added for Round 2	42/51 (82.3%)

Questions 12a, 13a, 14a, 15a, 19a, 23a, 24a, 26a, 30a, 31a, 32a represent modifications of the corresponding original questions and were introduced during the second Delphi round in response to feedback from panelists at the end of the first round of Delphi. The last 2 questions (34 and 35) were added to the second Delphi round, based on suggestions from panel members

**Box 1:** Consensus on the evaluation and management of patients undergoing transsphenoidal surgery

Item	
*Preoperative evaluation of pituitary function*	
Patients with an apparent pituitary adenoma should have serum prolactin measured preoperatively	
Serum IGF-I should be measured in all patients with a sellar mass preoperatively	
Patients with a macroadenoma or other large (≥ 1 cm) sellar mass should undergo evaluation for hypoadrenalism preoperatively	
Patients with a sellar mass who present with symptoms or signs suggestive of hypoadrenalism should undergo evaluation of adrenal reserve preoperatively regardless of lesion size	
All patients with an apparent pituitary adenoma and any symptoms or signs of hypercortisolemia should be evaluated for Cushing’s disease preoperatively	
Thyroid function should be tested in all patients with a sellar mass preoperatively	
Evaluation of gonadal function is advisable in patients with symptoms suggestive of hypogonadism, those with a large (≥ 1 cm) sellar mass and those with a functioning tumor regardless of size preoperatively	
Evaluation of possible diabetes insipidus is advisable in patients with a sellar mass who present with polyuria and/or hypernatremia preoperatively	
*Preoperative hormone replacement*	
Patients with hypoadrenalism should receive glucocorticoid replacement preoperatively	
Patients with hypothyroidism should receive thyroid hormone replacement preoperatively	
Stress dose glucocorticoid administration is generally advisable perioperatively in patients with known or suspected adrenal insufficiency	
*Postoperative endocrine evaluation*	
Serum sodium should be monitored postoperatively	
Morning serum cortisol should be monitored postoperatively (during an interval of 1–5 days postoperatively)	
Full evaluation of pituitary function should be conducted 6–12 weeks after transsphenoidal surgery	
Dynamic testing should be obtained to evaluate the pituitary adrenal axis at 6–12 weeks postoperatively, if morning serum cortisol is not sufficiently high to assure sufficient adrenal function early postoperatively	
Thyroid function should be assessed in all patients postoperatively (at 6–8 weeks)	
Gonadal function should be evaluated in patients (6–12 weeks) postoperatively, including women of premenopausal age and men	
Dynamic testing may be obtained to assess GH secretion postoperatively in patients with clinical suspicion of GH deficiency	
*Patients with acromegaly*	
Serum IGF-I should be obtained to evaluate endocrine remission at 6 weeks postoperatively. If elevated, serum IGF-I should be rechecked at 12 weeks postoperatively to document persistent disease activity before making treatment decisions	
Patients in endocrine remission should be evaluated biochemically for recurrence annually (or sooner if clinically indicated)	
*Patients with Cushing’s disease*	
Endocrine testing should be conducted during the first postoperative week to document endocrine remission	
Serum cortisol should be monitored to document endocrine remission postoperatively	
Patients in endocrine remission should be evaluated for recurrence annually (or sooner if clinically indicated)	
*Postoperative hormone replacement*	
Desmopressin may be administered “on demand” (as required) in patients who underwent transsphenoidal surgery and developed central diabetes insipidus in the postoperative period

**Box 2:** Items that did not achieve consensus after two rounds of the Delphi process

Item	
*Preoperative evaluation of pituitary function*	
Patients with an apparent macroadenoma and minimally elevated prolactin levels should have serum prolactin measured in serial dilution preoperatively	
*Patients with an apparent clinically non-functioning pituitary adenoma may be evaluated for Cushing’s disease preoperatively	
*Preoperative endocrine therapy*	
Sex steroid replacement is advisable in symptomatic patients with central hypogonadism preoperatively	
Sex steroid replacement may be considered in symptomatic patients with central hypogonadism preoperatively	
Preoperative medical therapy should be considered in patients with somatotropin-secreting adenomas	
*Preoperative medical therapy may be considered in patients with somatotropin-secreting adenomas	
Preoperative medical therapy should be considered in patients with corticotropin-secreting adenomas	
*Preoperative medical therapy may be considered in patients with corticotropin-secreting adenomas	
Stress dose glucocorticoid administration is advisable perioperatively	
*Postoperative endocrine evaluation*	
Patients with prolactinomas	
Serum prolactin should be measured postoperatively (on postoperative day 1 or 2) to evaluate for remission	
*Serum prolactin may be measured postoperatively (on postoperative day 1 or 2) to evaluate for remission	
All patients	
Dynamic testing should be obtained to assess GH secretion postoperatively in patients with clinical suspicion of GH deficiency	
Patients with acromegaly	
Morning serum GH should be obtained early postoperatively (postoperative day 1 or 2) to evaluate endocrine remission	
Morning serum GH may be obtained early postoperatively (postoperative day 1 or 2) to evaluate endocrine remission	
A glucose tolerance test should be obtained to evaluate endocrine remission several weeks postoperatively	
*A glucose tolerance test may be obtained to evaluate endocrine remission several weeks postoperatively	
Patients with Cushing’s disease	
Monitoring of plasma ACTH levels should be considered to document endocrine remission postoperatively	
Monitoring of plasma ACTH levels may be considered to document endocrine remission postoperatively	
24 h urinary free cortisol should be monitored to document endocrine remission postoperatively	
*24 h urinary free cortisol may be monitored to document endocrine remission postoperatively	
Late night salivary cortisol should be monitored to document endocrine remission postoperatively	
*Late night salivary cortisol may be monitored to document endocrine remission postoperatively	

*Consensus (≥ 80%) would be achieved on these items if responses from panelists with any extent of agreement (ratings 5, 6, 7 on the Likert-type scale) were considered

### First Delphi round

Delphi panel members were provided with an electronic link to the online questionnaire. For each item, the panelists were asked to rate their agreement or disagreement on a Likert-type scale as follows: 1 (“complete disagreement”), 2 (“some disagreement”), 3 (“disagreement”), 4 (“neither disagreement nor agreement”), 5 (“agreement”), 6 (“some agreement”) to 7 (“complete agreement”). Panel members were allowed to skip any questions they did not wish to answer. In addition, panelists were provided with the opportunity to suggest additional questions or make comments. Panel members were given 4 weeks to complete the survey, and a single reminder was sent to those who did not respond. Of 55 invited panelists, 50 responded in the first round. Anonymized data from the first round were summarized and sent to all participants. In addition, several questions were modified and additional questions were added by the research team, based on comments and suggestions made by panel members in the first round.

### Second Delphi round

Panel members were recontacted via electronic mail and 51 of the originally invited panelists participated in the second round. Anonymized feedback, showing the distribution of panelists’ agreement with each item from the first round, was provided to all Delphi panel members in the second round.

Panelists were then asked to rate their agreement with each item using the same Likert-type scale used in the first round. Questions that had reached strong consensus (defined as ≥ 80% of participants rating their agreement as ≥ 6 or ≤ 2 on the Likert-type scale) during the first round were not included in the second Delphi round. Instructions given to panel members were otherwise identical to those provided during the first Delphi round.

A virtual meeting was originally conceived as a means of building further consensus in a modified Delphi process. However, consensus was achieved for the majority of items by the end of the second round, with only modest changes in agreement among panelists between the two rounds of Delphi. Due to COVID-19 restrictions and strenuous demands on healthcare professionals in many of the countries represented in the Delphi panel, a virtual meeting after the second round of Delphi was not convened.

### Data analysis

The proportion of panelist consensus across each item was tabulated based on predefined criteria (a response of ≥ 6 signified agreement and ≤ 2 signified disagreement with statement). The proportion of items that reached consensus was tabulated, as was the proportion of statements that achieved consensus after question modification.

## Results

Thirty-five items pertaining to the preoperative and postoperative endocrine evaluation and management of patients with sellar masses undergoing transsphenoidal surgery were prepared by the Steering Committee. These items were subsequently submitted to the Delphi panel for consideration, including two items that were introduced in the second round of Delphi in response to feedback from panelists at the end of the first round.

The proportion of Delphi panelists that indicated some or complete agreement with each item (rating 6 or 7 on a Likert-type scale) is shown for each of the two rounds of Delphi (Table 1). A high proportion of panelists (range 46–51 of 51 participants) provided ratings for individual items. Using our prespecified criteria (≥ 80% of panelists rating their agreement as ≥ 6 or ≤ 2 on the Likert-type scale), consensus was achieved for 24 of 35 items (68.6%) at the end of the second Delphi round, including three items for which consensus was reached after modification or addition of questions for the second round.

There was general agreement amongst panelists with respect to preoperative evaluation of pituitary function, with consensus achieved for 8 of 9 items (88.9%). On the other hand, there was less agreement amongst panelists regarding preoperative therapy [3 of 6 items (50%)]. Regarding postoperative endocrine evaluation, consensus was achieved for 12 of 19 items (63.2%). Consensus was also reached on the single item on postoperative therapy (1 of 1 item). Items for which consensus was reached are summarized in Box 1.

Using predefined criteria on panelists’ extent of agreement (Box 2), consensus was not achieved for 11 items after two rounds of Delphi. If using less stringent criteria of agreement (i.e., ratings of ≥ 5 on the Likert-type scale), similar to other Delphi panels [13, 15], consensus would be reached for 88% of items (31 of 35 items, Box 2).

## Discussion

In this study, a Delphi process was used towards building consensus on endocrine aspects of perioperative evaluation and management of adults undergoing transsphenoidal surgery [1, 2].

After two rounds of Delphi, panelists from Asia, Europe, North America, Oceania, and South America reached consensus on the majority of items submitted to them by the research team. There was wide agreement on the extent of preoperative and postoperative endocrine testing. Despite some uncertainties in previous guidelines [16], a clear consensus was achieved in this group to measure serum IGF-I in all pituitary tumors preoperatively to ensure proper diagnosis of GH excess. This is important because patients with GH-secreting adenomas do not always present with classic manifestations of acromegaly, require additional evaluation for comorbidities and may benefit from further medical therapy [17].

There was agreement on preoperative administration of glucocorticoid and thyroid hormone replacement in patients with diagnosed deficiencies as well as perioperative use of stress-dose glucocorticoid coverage for patients with known or suspected hypoadrenalism, but not for all patients undergoing transsphenoidal surgery. The panelists also agreed on postoperative monitoring of serum sodium and cortisol and the use of desmopressin “on demand” (as required to control hypernatremia and/or polyuria) for patients with central diabetes insipidus. Agreement was achieved on postoperative monitoring of endocrine function, including morning serum cortisol in patients with Cushing’s disease, as well as serum IGF-I in patients with acromegaly.

Regular monitoring of serum sodium levels is important and advised for approximately 7–10 days postoperatively, including after hospital discharge. This will detect the possibility of hyponatremia, which could be as high as 20%, with symptomatic cases in just 5% of these patients or more rarely, a trend for triphasic response. These items are broadly consistent with several published guidelines [5–7, 18] and highlight the importance of guideline practicability, even when the level of evidence per se is limited in real-world scenarios.

On the other hand, there were several items for which consensus was not achieved by the Delphi panel, using our strict, predefined criteria. This at least partly reflects those topics where there are either no data available or where there is controversy in the literature and more research is needed.

Notably, panelists did not broadly agree on the need to measure serum prolactin in dilution in patients with macroadenomas. Prolactin immunoassays can be susceptible to the “hook effect” artifact, which may lead to substantial underreporting of prolactin measurements in sera containing very high prolactin concentrations [19, 20]. Although rare, this artifact can lead to misclassification of a prolactin-secreting adenoma as a non-functioning lesion, thus having important implications for patient management. Some panelists commented that they would recommend measuring prolactin in dilution only in sera from patients with giant adenomas, while others noted that their laboratory routinely tests for deviations from linearity of measured prolactin, using automated assay platforms (data not shown). While newer automated immunoassay platforms are likely to detect the “hook effect”, this may not be the case in older assays, which are still in use in many countries. Therefore, there is potential for misdiagnosis [21, 22], especially when surgery is performed at an institution where automated assays are available to detect hook effect, yet patient workup has been carried out at an outside laboratory that does not use such technologies [23]. This scenario can occur more often with the widespread use of telemedicine, including both distance visits and endocrine testing being carried out at a distant laboratory. Careful communication between the clinician and laboratory staff is advised to minimize this possibility.

There was also lack of consensus regarding preoperative testing for hypercortisolism in all patients with an apparently non-functioning pituitary adenoma. This might reflect concern about false positive results of endocrine testing in some individuals. On the other hand, published data suggest that some patients with Cushing’s disease may lack typical symptoms and signs, and can present with an incidentally found sellar mass [24]. These patients are at risk of developing severe hypoadrenalism postoperatively if glucocorticoid replacement is not instituted. In addition, silent corticotropinomas cannot be reliably diagnosed in the absence of preoperative testing for hypercortisolism [25, 26]. Such patients seem to have a worse prognosis [27, 28].

Preoperative sex steroid replacement was not agreed upon, even in symptomatic patients with hypogonadism. Some panelists raised concerns that sex steroid administration carries a prothrombotic risk, which may be even higher in the perioperative period (data not shown) [29]. There are no data suggesting a better surgical outcome if sex steroids are replaced preoperatively.

Items concerning the administration of preoperative medical therapy in patients with acromegaly or Cushing’s disease did not reach consensus, potentially reflecting differences in practice among international centers (including various waiting times between diagnosis and transsphenoidal surgery among different centers), the clinical heterogeneity of patient populations, and ongoing uncertainties regarding the benefits of preoperative medical therapy [30–32]. Clinical experience suggests that preoperative medical therapy may be helpful in patients with Cushing’s disease and very high cortisol levels who present with acute psychiatric illness or sepsis.

Interestingly, despite substantial work in this area, the definition of remission in all hyperfunctioning pituitary tumors remains controversial, including the role of immediate postoperative laboratory testing [10, 12]. Several items pertaining to the postoperative assessment of remission in patients with functioning tumors did not achieve consensus, including early prolactin or growth hormone (GH) measurements in patients with prolactinomas or acromegaly, respectively, and subsequent post-glucose GH testing in acromegaly, or the measurements of plasma adrenocorticotropic hormone, 24-h urinary free cortisol, and late-night salivary cortisol in Cushing’s disease. These observations may partly reflect differences in practice among centers or perhaps lack of clinician confidence with regards to the accuracy of some endocrine assays. Some data support the predictive value of these tests in assessing endocrine remission [33–36]. Additional research would be helpful to inform expert opinion and clinical practice pertaining to assessment of endocrine remission.

Strengths of the present study include the relatively large size of the Delphi panel, the inclusion of panel members from an international pool of experts from five continents, including a majority from institutions fulfilling criteria for Pituitary Centers of excellence [37], and the very high proportion of panelists who engaged effectively in the Delphi process by rating each item. The present report represents real-life experience, reflecting variations in the approach to endocrine management in different parts of the world among diverse healthcare systems and varying financial resources.

In conclusion, substantial consensus on endocrine aspects of the perioperative evaluation and management of adults undergoing transsphenoidal surgery was reached, using a Delphi process. However, consensus was not reached on a minority of items, some of which represent areas where further research is needed to inform expert opinion, pituitary care and further consensus guidelines.

## Data Availability

The dataset can be made available upon reasonable request.
